# Hypercoagulation and elevation of blood triglycerides are characteristics of Kawasaki disease

**DOI:** 10.1186/s12944-015-0167-2

**Published:** 2015-12-30

**Authors:** Xi Chen, Zhen-Wen Zhao, Lin Li, Xue-Jun Chen, Hui Xu, Jin-Tu Lou, Lin-Jie Li, Li-Zhong Du, Chun-Hong Xie

**Affiliations:** Department of Cardiology, Children’s Hospital, Zhejiang University School of Medicine, No.3333 Bin-Sheng Road, Bin-Jiang Dist, Hangzhou, Zhejiang 310052 China; Key Laboratory for Diagnosis and Treatment of Neonatal Diseases of Zhejiang Province, Hangzhou, Zhejiang China; Beijing National Laboratory for Molecular Sciences, Key Laboratory of Analytical Chemistry for Living Biosystems, Institute of Chemistry, Chinese Academy of Sciences, Beijing, China

**Keywords:** Kawasaki disease, Lipidomics, Triglyceride, Plasma, Serum, Hypercoagulation, Thromboelastography

## Abstract

**Background:**

Cardiovascular damages poses risks to children with Kawasaki disease (KD). Although hypertriglyceridemia and hypercholesteremia are risk factors of cardiovascular damages, studies on the blood lipid metabolism in KD are still limited. This study aims to analyze the blood lipids and coagulation in KD.

**Methods:**

Triglyceride (TG) and cholesterol levels in the plasma and serum from 20 children with KD were examined in comparison with 10 healthy children (HC) as well as 10 children with high fever from identified bacterial infections (BT). Using electrospray ionization mass spectrometry, we profiled the lipid species. Blood coagulation was analyzed. Statistics was analyzed by one-way ANOVA using SigmaStat.

**Results:**

We found that in KD, plasma TG level was significantly increased, but not serum TG. A total of 19 molecular species of TG were identified, and they were all increased in KD and BT patients, and more pronounced in KD. On the other hand, major molecular species of plasma phosphotidylcholine and lyso-phosphotidylcholine were decreased in KD and BT. Pronounced hypercoagulation was found in KD blood.

**Conclusion:**

Our data indicate hyperlipidemia in KD, especially for TG, which contributes to the hypercoagulation and the potential risk of cardiovascular damages. Evaluation of blood lipid levels in severe KD patients could provide valuable information for treatment and prognosis, thus would be worthy of consideration.

**Electronic supplementary material:**

The online version of this article (doi:10.1186/s12944-015-0167-2) contains supplementary material, which is available to authorized users.

## Background

First described by T. Kawasaki [1], Kawasaki disease (KD) is a systematic syndrome with unknown etiology. It broadly affects blood vessels, skin, mucous and lymph nodes, and is typically manifested by fever and systemic vasculitis, and poses risk to patients’ life when the coronary arteries are affected. KD is most common in children under 5 years of age, males and of Asian ethnicity. It is attributed to immune disease since KD vaculitis is accompanied by increase of inflammatory cells and cytokines without identified pathogens, and is responsive to intravenous immunoglobulin and corticosteroid therapy. In our provincial children’s hospital in Zhejiang, China, about 350 KD patients are admitted each year and 5-6 % are accompanied by coronary artery lesions, while severe cardiovascular damages including and valvular insufficiencies and cardiogenic shock are rare complications [2]. Intravenous immunoglobulin is our first line therapy with the responsive rate of 85 %, yet the risk to develop cardiovascular damages is increased in intravenous immunoglobulin-resistant patients. The recovered KD patients have a higher risk to develop atherosclerosis in adult years [3–6].

Although the accompanying and secondary cardiovascular damages pose great risk to KD children, studies on the blood lipid metabolism in KD are still limited. Biolipids are not only compositional materials as compartmental barrier, but also signaling molecules that mediates cellular functions. Lipid metabolites are currently used as biomarkers for the diagnosis and prognosis of cardiovascular diseases, cancer, and neurological diseases. In recent years, tandem mass spectrometric technique has greatly facilitated the quantitative and qualitative studies of biolipids, and lipidomics analysis is currently being expanded as a diagnostic tool for the benefit of personalized medicine [7]. The blood contains thousands of biolipid species, the levels are determined by both food intake and acquired or secondary factors including individual genetics and metabolic traits. In this study, we analyzed the blood lipid profile in KD children in comparison with healthy children (HC) and children with high fever (>38 °C) from diagnosed bacterial infections (BT) from the same age group by biochemical methods as well as electrospray ionization mass spectrometry (ESI MS).

Blood triglyceride (TG) is distributed between chylomicrons and very-low density lipoprotein. It has been recognized that hypertriglyceridemia is a risk factor of cardiovascular damages independent of cholesterol [8–12]. Our study has analyzed the specific change of TG molecular species as it is the major component of blood lipids. There is a potential link established between increased blood TG and low-grade inflammation [10, 12] that is also manifested in KD patients.

## Results

### Hypercoagulation in KD patients

KD patients were diagnosed based on symptoms [1, 13]. On the day when fasting blood samples were collected, the patients were still feverish, with C-reaction protein (CRP) and erythrocyte sedimentation rate (ESR) significantly exceeding the normal range (Table 1), suggesting persisted inflammation.

**Table 1:** Summarized clinical information of the 20 patients with Kawasaki disease recruited in our study on the day of blood sample colllection

	Age	Sex	Days of fever	Body temp, °C	CRP^a^	ESR^b^
1	4y9m	M	3	38.8	83	66
2	5y1m	F	5	38.5	94	64
3	4m23d	M	5	36.8	110	62
4	5y6m	F	7	36.8	>160	75
5	8m13d	M	8	39.0	23	76
6	3y10m	F	5	38.5	128	93
7	2y9m	M	5	39.5	63	66
8	1y1m	M	5	38.0	139	66
9	3y11m	F	5	39.1	98	88
10	5y2m	M	5	38.5	41	55
11	1y8m	M	6	39.5	69	55
12	1y6m	F	6	38.9	>160	57
13	1y10m	M	3	37.2	65	40
14	1y5m	M	5	40.5	20	35
15	2y	F	3	39.1	>160	73
16	5y4m	M	4	38.8	95	75
17	6m17d	M	3	39.0	25	83
18	1y5m	M	4	38.5	40	62
19	1y2m	F	3	38.8	45	87
20	3m22d	M	4	39.0	29	66

^a^C-reaction protein (CRP), normal range 0-8 mg/L

^b^erythrocyte sedimentation rate (ESR), normal range 0-20 mm/h

**Fig. 1:**
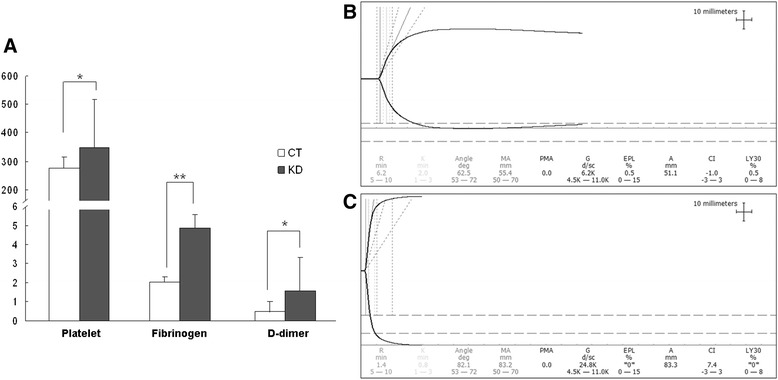
Blood coagulation and thromboelastography analysis. **a** Comparison of platelet number, plasma fibrinogen (g/L) and D-dimer (mg/L) levels from healthy children (HC) and Kawasaki disease (KD). *: *P* < 0.05. **: *P* < 0.01. **b-** **c** Representative thromboelastographs from blood samples with normal coagulation (**b**) and with hypercoagulative state in a patient with Kawasaki disease (**c**)

From Fig. 1a, the platelet number was 349 ± 169 × 10^9/L in the KD group, and 276 ± 39 × 10^9/L in the group of healthy controls (HC). The fibrinogen level was 4.88 ± 0.71 g/L in the KD group, and was 2.04 ± 0.48 g/L in the HC group. The level of D-dimer was 1.55 ± 1.74 mg/L in the KD group, and was 0.48 ± 0.52 mg/L in the HC group. The statistical differences were significant (*P* < 0.05). Prothrombin time (PT), activated partial thromboplastin time (APTT) and thrombin time (TT) were all within the normal range and showed no significant differences between all sample groups (Additional file 1: Table S1).

Thromroelastography (TEG) monitors the process of blood clot formation and retraction dynamically. It was first established by Harter in 1948, and has been proved valuable in examining coagulation specifically [14, 15]. Our facility produces the following parameters of significance: R (reaction time) stands for the time for initial fibrin formation, and prolongation of R value is due to coagulation factor deficiencies; K (coagulation time) measures the speed of clot strengthening, and increased K value suggests hypofibrogenemia or platelet dysfunction; the maximum amplitude (MA) represents the clot strength, and is a parameter to judge platelet function; the coagulation index (CI) is a parameter for the overall coagulation state. We examined 20 KD blood samples by TEG, and the results have revealed marked hypercoagulation. Typical thromboelastographs from a healthy individual and that from a KD patient are shown (Fig. 1b, c). As summarized in Table 2, in the KD patients, the R value was significantly lowered (4.3 ± 1.8 compared with 5-10), the K value was within the normal range, MA was significantly increased (75.9 ± 4.6 compared with 50-70), and so was CI (3.9 ± 1.8 compared with -3- + 3). As judged by CI, the occurrence rate of hypercoagulation in KD was 80 %. Most of the KD patients had abnormal MA (95 %) and R (80 %) values, while only 15 % had abnormal K.

**Table 2:** Collected parameters by thromboelastography analysis of blood samples from 20 patients with Kawasaki disease

	R	K	MA	CI
Mean	4.3	1.2	75.9	3.9
SD	1.8	0.3	4.6	1.8
Reference range	5—10	1—3	50—70	-3—3
Abnormal rate	80.0 %	15.0 %	95.0 %	80.0 %

### Increased plasma TG, but not serum TG in KD

**Fig. 2:**
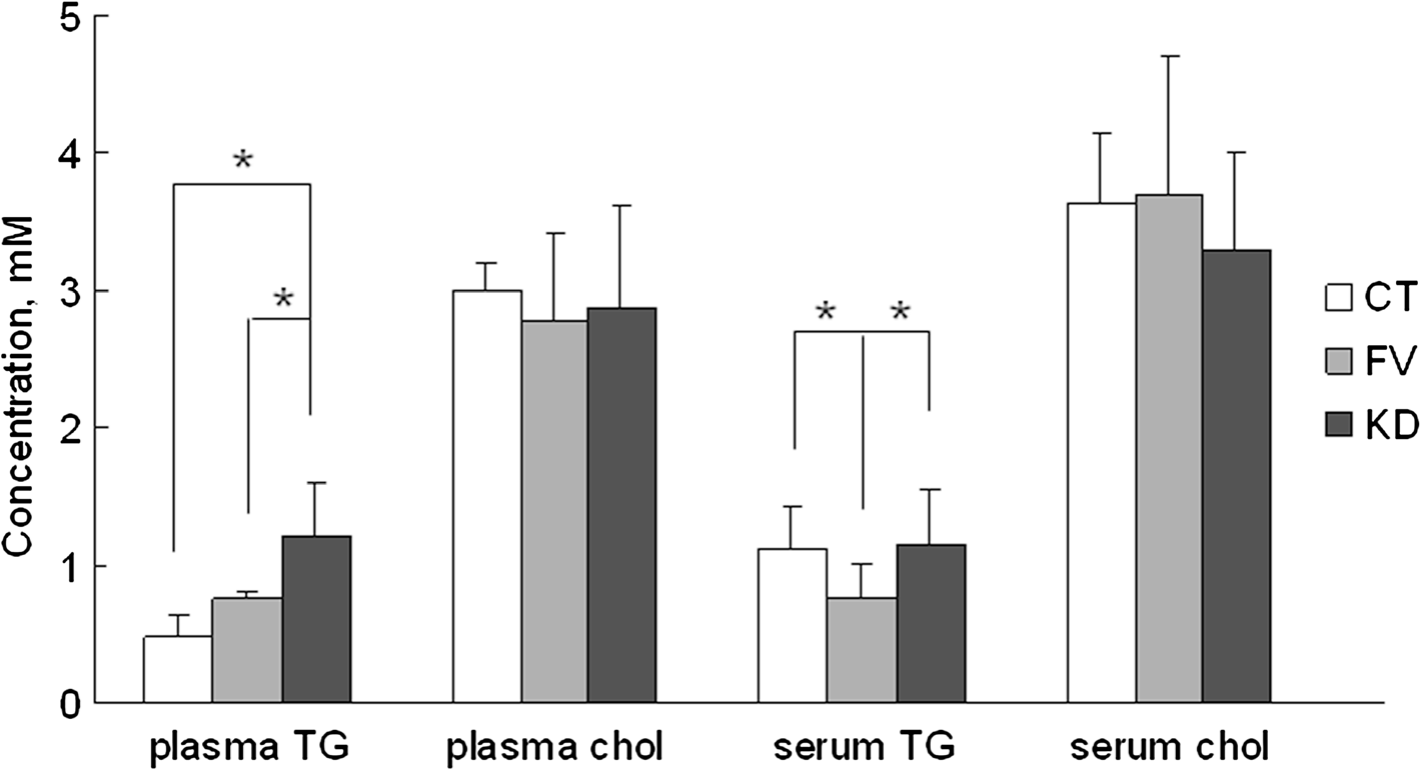
Serum and plasma triglyceride and cholesterol levels. Blood biochemistry analysis of plasma and serum triglyceride (TG) and cholesterol (chol) levels from 3 groups: healthy children (CL), patients from diagnosed bacterial infections (BT) and patients with Kawasaki disease (KD). *P: <0.05

Both plasma and serum are being used for blood lipid testing. From the literature, the plasma is considered to be more optimal to reflect the original physiological properties compared with the serum [16]. TG and cholesterol levels from serum and plasma were both examined. As shown in Fig. 2, total plasma TG was significantly increased in KD compared with the group of HC and the feverish children with bacterial infections (BT). Levels of plasma TG in BT and HC groups were not statistically significant. However, examination of the serum revealed a different scenario. The TG levels in the HC and KD groups were not statistically significant, while it was significantly decreased in BT patients. Cholesterol levels were similar in all 3 groups, both in the plasma and the serum.

### Plasma TG profiling by mass spectrometry

**Fig. 3:**
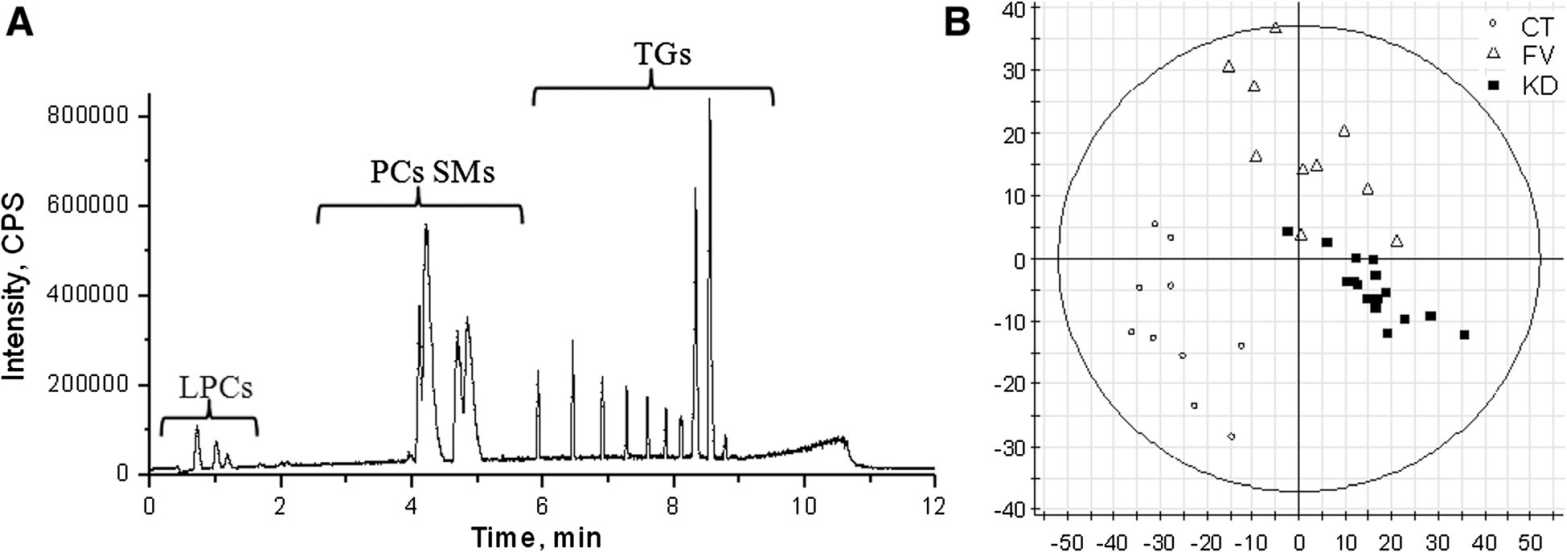
Detection of polar lipids by UPLC-ESI-MS and discriminant analysis between groups. **a** Total ion current of representative plasma lipid extract from UPLC-ESI-QTOF MS. Within the 12 min of elution time of each sample, lyso-phosphatidylcholine (LPC), phosphatidylcholine (PC) and sphingomyelin (SM) were eluted serially, followed by triglyceride (TG). **b** The PLS-DA scoring chart by EZ info software of plasma lipids detected by the positive ESI mode MS from three groups (HC: control; BT: feverish with bacterial infections; KD: Kawasaki disease) indicates separation of sample groups

Our established high performance liquid chromatography-electrospray ionization- quadrupole time of flight mass spectrometry (HPLC-ESI-QTOF MS)-based protocol scans over 20,000 molecular species of polar lipids. All lipids were eluted from the HSS T3 column within 12 min of elution (Fig. 3a), lyso-phosphatidylcholine (LPC) species were eluted at the first 2 min, phosphatidylcholine (PC) species were eluted from 2 to 6 min, while TGs were eluted from 6 to 10 min. By performing partial least square-discriminant analysis, the 3 experimental groups were well separated (Fig. 3b) and lipid species contributing to the discriminative separation were picked and confirmed by LIPID MAPS. Besides the neutral lipid cholesterol, the major plasma lipid is TG and PC. Consistently, we found these two classes were the major contributing species to discriminate the separation between the 3 groups.

**Fig. 4:**
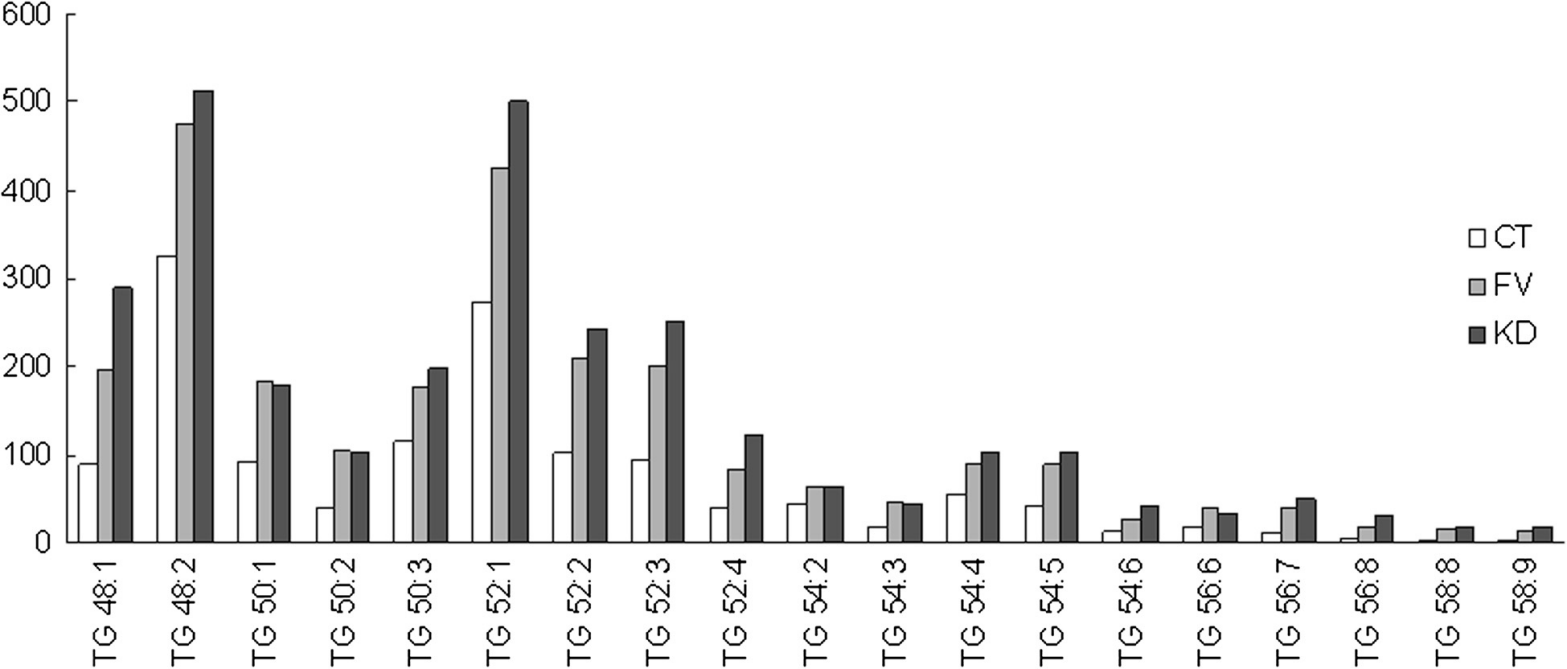
Plasma triglyceride profiling and quantification. Profiling and quantification of triglyceride (TG) molecular species from the plasma of healthy children (HC), children with fever from bacterial infections (BT) and Kawasaki disease (KD). Y axis indicates the area after normalization from Q-TOF MS, a relative quantification instead of direct measurements of the absolute quantity. Data are presented as mean values, SD and P values are presented in Additional file 1: Table S2

From the most abundant 100 molecular species of plasma polar lipids, 19 was identified as TGs, which were all significantly increased in KD as well as BT patients (Fig. 4 and Additional file 1: Table S2). From the 19 TGs, 5 species were significantly increased in KD compared with the BT group: TG 48:1, TG 52:1, TG, 52:3, TG 52:4 and TG 56:8. These are the relatively more abundant TGs, consisting 41 % of total TG in KD, and 36-37 % in HC and BT groups.

### Decrease of plasma PC and LPC in KD patients

**Fig. 5:**
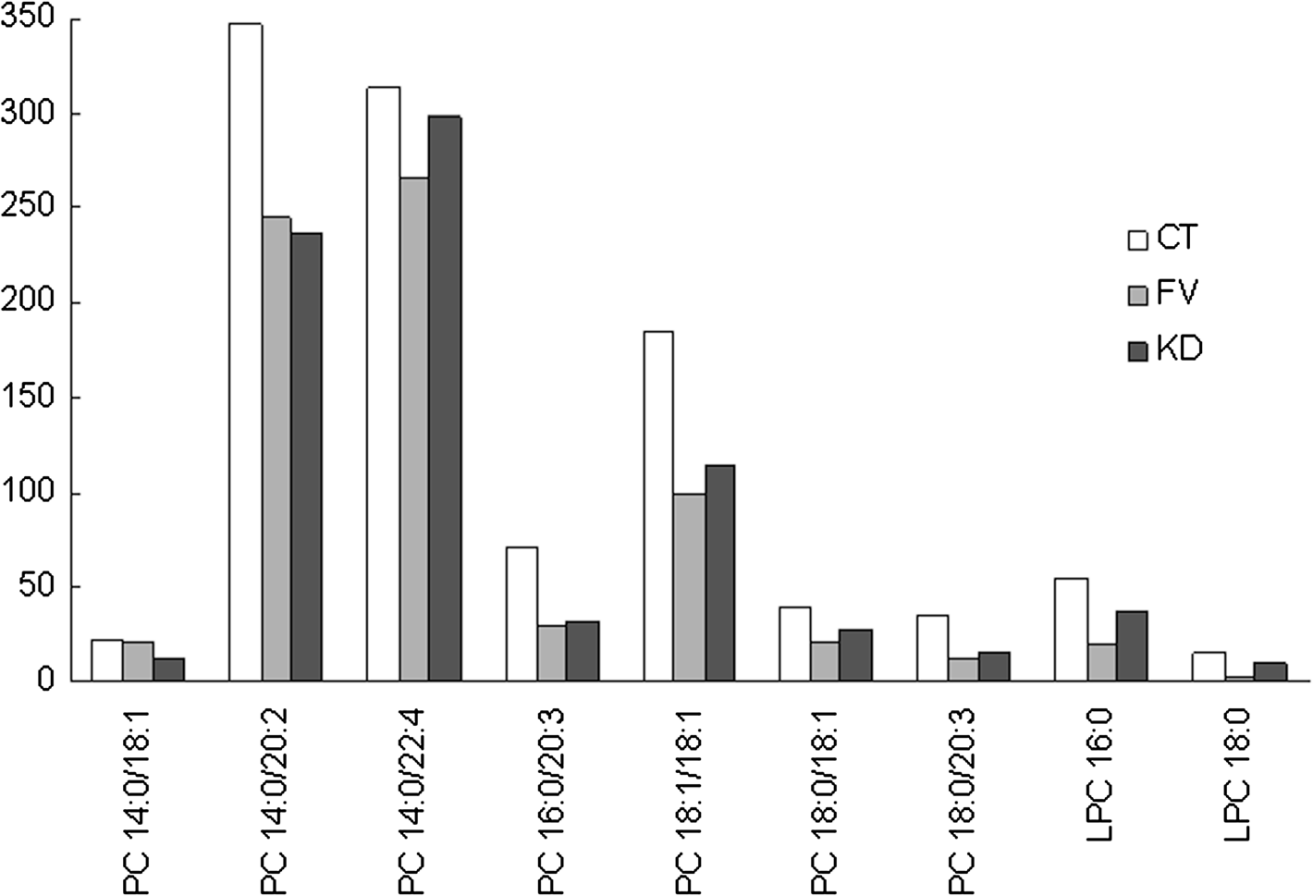
Profiling and quantification of plasma phosphatidylcholine and lyso- phosphatidylcholine. Profiling and quantification of major phosphatidylcholine (PC) and lyso- phosphatidylcholine (LPC) molecular species from the plasma of healthy children (HC), children with fever from bacterial infections (BT) and Kawasaki disease (KD). The Y axis indicates the area after normalization from Q-TOF MS. Data are presented as mean values, SD and P values are presented in Additional file 1: Table S3

PC is the second abundant group of polar plasma lipids. In our study, we have observed significant changes in several PC molecular species from KD and BT groups compared with controls, including 7 PCs and 2 LPCs. Contrary from TG, PC and LPC species were decreased in patients (Fig. 5 and Additional file 1: Table S3). Of the 7 PCs of interest, 2 molecular species with relatively shorter free fatty acid (FFA) chains, PC 14:0/18:1 and PC 14:0/20:2, were more significantly decreased in KD, while the other 5 were more significantly decreased in BT. However, change of PC profile was not significant between KD and BT groups. Change of the two LPCs was more striking than PC, significantly decreased in KD and even further decreased in BT (Fig. 5 and Additional file 1: Table S3). It was possible to separate KD and BT by the LPC content because of the extremely sharp decrease of LPC in BT patients.

## Discussion

### Hypercoagulation in KD and anti-coagulation therapy

Platelet counting and coagulation analysis revealed significant increase of plate number and levels of fibrinogen and D-dimer in KD (Fig. 1a), suggesting hypercoagulation as a common complication of KD patients. As a point-of-care testing method, TEG monitors the dynamic formation of blood clots, and is advantageous than traditional measurement of coagulation factors in its fast testing speed and the ability to further discriminate between platelet- and fibrinogen-associated coagulation. Although no increase of prothrombin time, activated partial thromboplastin time and thrombin time was detected, TEG indicates that blood coagulation was faster in 80 % KD patients (Table 2). Change in MA that represents platelet dysfunction was manifested in 95 % of KD patients, while the K value associated with fibrinogen was mostly within the normal range. Based on our data and previous clinical experience, increased fibrinogen level does not necessarily lead to significant fastened blood coagulation. Fibrinogen is an acute-phase protein produced in the liver upon stimulation of a broad spectrum of stress signals including inflammation, trauma, etc. Compared with the fibrinogen level, activation of platelet is a more significant contributing factor of fastened blood coagulation. Both PT/APTT and TEG analysis suggested that hypercoagulation in KD patients was mainly attributed to platelet over-activation, although hyperfibrinogenemia was also involved.

Previous retrospective research identified secondary thrombocytopenia as a complication in some KD patients [17, 18], and is a risk factor for severe cardiovascular damages including coronary artery aneurysm and acute myocardial infarction [19, 20]. These studies also suggested that thrombocytopenia in KD was a result of intravascular coagulation [20]. Furthermore, although not occurred in our hospital, literature reported a couple of KD cases with a rare but highly dangerous complication: disseminated intravascular coagulation (DIC), the dysregulated balance between coagulation and fibrinolysis [21, 22]. DIC could occur in KD patients without evidence of pathogen invasion. Based on our findings and the literature, we advise the clinicians to be cautious with the hypercoagulative state in KD patients, especially for the severe and intravenous immunoglobulin-resistant cases to prevent serious complications. Guided anti-coagulation therapy could be of benefit to patients with positive laboratory findings and other risk factors.

### Abnormal plasma TG in KD children

We have examined the levels of TG and cholesterol from both serum and plasma, and found plasma TG levels were significantly increased in KD compared with HC and BT groups, while there was no difference in the cholesterol levels (Fig. 2). TG is the major component of very-low density lipoprotein and chylomicrons, while cholesterol is mainly transported in low density lipoprotein. Cholesterol and low density lipoprotein are long considered as a risk factor for cardiovascular damages, but the pathological role of TG and very-low density lipoprotein in cardiovascular damages has only been revealed in recent years. TG hydrolysis in arterial wall foam cells could lead to development of atherosclerosis plaques [11]. Lipolysis of TG-rich lipoproteins produces potentially toxic oxidized FFAs, monoacylglycerols and other molecules to activate immune cells and stimulate the production of cytokines along the artery wall [23, 24]. Genome-wide association studies also suggested causal association between elevated TG and cardiovascular damages [25, 26]. Thus increased blood TG levels could exacerbate inflammation and arterial damage. Our data suggested that plasma TG metabolism is a more sensitive parameter than cholesterol in the sterile inflammation of KD compared with bacteria-induced infection. Such lipid metabolic change partly explains why KD patients have extremely high risks to develop cardiovascular damages such as arteriosclerosis. Consistently, in recent publications, increase of serum TG (plasma not examined) in late stage KD was discovered [27], and use of statins was shown to benefit KD patients to reduce the inflammation and the endothelial function [28]. In current clinical practice, both plasma and serum are used to perform blood lipid analysis. Although there is still no general guidance in discriminating the two categories of samples in blood lipid testing, our data suggest that blood coagulation may affect the lipid profile, such as TG, thus plasma could be a more reliable object as other researchers have reported [16].

During our study, we found that the TG levels were clinically reported normal in all patients, however, our comparative analysis revealed statistic difference between HC, KD and BT children. This is watchful that the current blood lipid reference range in pediatric laboratory medicine may not be suitable, at least not for the Chinese children under 5 years of age targeted by our research. In detail, our currently applied upper value for plasma cholesterol is 4.4 mM, and is 1.5 mM for TG, lower than the standards applied for adults (5.2 mM for cholesterol and 1.7 mM for TG). Our clinical reference range (plasma cholesterol < = 4.4 mM, plasma TG < = 1.5 mM) is slightly lower than that applied in adults, either comparable [29] or not comparable [30, 31] with data obtained from same age group of children from other countries. Considering that study on the epidemiology of hypertriglyceridemia in Chinese children is still very limited in number, we advice re-evaluation of the current standards by further larger-scale analysis to better discriminate the healthy and diseased states in blood lipid metabolism during childhood.

### Profiling of plasma TG molecular species in Chinese children by ESI MS lipidomics analysis

Instead of exhaustive identification of all lipids as some researchers [32, 33], we selected the lipid species contributing to the difference between the three sample groups from the top 100 abundant species. 19 TG species were the most abundant in prevalence and the most significant contributing components to differentiate the 3 sample groups (Fig. 4). All the 19 TG species were increased in KD and BT patients, and more significantly in KD, further supporting the increase of the absolute TG level in the diseased conditions and a general malregulation of TG metabolism. From our data, in Chinese children under 5 years of age, the most dominating TG species were TG 48:2, TG 52:1, TG 50:3, TG 52:2, TG 52:3, TG 50:1 and TG 48:1, contributing to over 70 % of total TG (Additional file 1: Table S1). Palmatic acid (16:0) and oleic acid (18:1) were the most common FFA chains in identified TGs, and the average total carbon number was under 54. This is less variable and much longer than the reported result based on the elder western populations [16, 34]. The FFA composition is determined by both diets and the metabolic mechanisms, both should contribute to the difference between our target population and others’.

In 2014, several reports were published based on a large Cohort study named San Antonio Family Heart Study covering over 1200 adult Mexican Americans. Plasma lipid composition was found to be inheritable and associated with risk of cardiovascular damages [35, 36]. In these studies, 43 TG molecular species were identified by ESI MS, and the statistical distribution of various species was associated with age, sex and body mass index [37]. In another recent study with the intention to identify lipid biomarkers for cardiovascular damages [34], shotgun lipidomics detected 135 lipid species, among which TG 54:2 has been found to be a risk factor. Certain species of TG were attributed to childhood obesity [38] and insulin resistance [39]. Our analysis of molecular species of TG in the blood suggest metabolic disorder of TG in both bacteria-induced and sterile inflammation, especially the latter. Further enlarged samples may be helpful to identify whether it is a risk factors for KD-associated cardiovascular damages. Discovery of novel lipid biomarkers will facilitate diagnosis of severe complications of KD, and to discover more therapeutic targets in the future.

Besides increase of TG, we also found marked decrease of PC and LPC in patients. Unlike TG, change of PC and LPC was more pronounced in the fever group caused by bacteria infection (Fig. 5). Our fever group is composed of children with pneumonia, and in our hospital, such cases are mostly caused by *S. pneumoniae*, other pathogens include *haemophilus influenzae*, *mycoplasma pneumoniae* and *Klebsiella pneumoniae* (intercommunications). The underlying mechanism of TG and PC/LPC malregulation remains to be discovered. In KD patients, blood cytokines levels including IL-1, IL-6, IL-10, TNFα, and BTNγ were significantly increased [40, 41]. There was laboratory evidence showing TNFα, IL-1 and other cytokines could induce TG synthesis [42–44], meanwhile, association of cytokine patterns with TG levels have been observed in human [45, 46]. Research on the regulation of inflammatory factors on lipid metabolism will be informative. Based on previous literature reports, PPARγ was increased in KD and could be a potential anti-inflammatory candidate target for cardiovascular damages in KD cases [47]. It remains to be confirmed if PPARγ could serve as a promising therapeutic target for KD.

In summary, our research has identified pronounced change in plasma TG, PC and LPC in KD patients with detailed profiling of molecular species. Increase of blood TG, together with hypercoagulation in KD imply the necessity to control blood lipid levels as well as anti-coagulation therapy, such as use of statins.

## Conclusions

Our study demonstrated hyperlipidemia in KD children, especially for TG species, which contributes to hypercoagulation and the potential risk of cardiovascular damages, thus would be worthy of further evaluation in severe KD patients to provide information to guide treatment and prognosis. Meanwhile, our data suggested malregulation of blood lipid metabolism in KD, and should be subjected to further investigations.

## Methods

### Patients and controls

This study was approved by the ethics committee of the Children’s Hospital, Zhejiang University School of Medicine. The study protocol conforms to the ethics guidelines of the 1975 Declaration of Helsinki, as was reflected in the a priori approval provided by the institution’s human research committee. Informed consent was obtained from each patient’s parent or guardian.

Fasting blood samples were collected from 20 KD patients, 10 patients of bacterial pneumonia with body temperature above 38 °C (bacterial infection group, BT), and 10 healthy children for routine body examination (healthy control group, HC). All patients were admitted to the hospital ward from June to October, 2013. The KD patients were 13 boys and 7 girls aged between 4-month to 5-year-6-month, the median age was 2-year-9-month. At the time of blood collection, all KD patients were in the acute stage with fever above 37 °C for over 3 days and without intravenous immunoglobulin administration, which was proved to be effective to all cases in the later treatments. The clinical information of KD patients is summarized in Table 1.

### Processing of blood samples, blood biochemistry and thromboelastography analysis

Serum samples were subjected to laboratory test for CRP, ESR, TG/cholesterol levels and other parameters by an automatic biochemistry analyzer (DPP modular, Roche, USA). Blood samples with the presence of sodium citrate as anti-coagulant were analyzed by an automatic blood analyzer (Mindray BC-5300, China) for the platelet number, etc. Levels of fibrinogen and D-dimer were analyzed by a full-automatic coagulation analyzer (Sysmex CA1500, Japan).

Thromroelastography (TEG) was collected from a TEG5000 Haemoscope (Niles, USA), with sodium citrate as anti-coagulant and kaolin as coagulation initiator. In TEG analysis, 1 mL whole blood is placed in a rotating metal cuvette heated to 37 °C, as fibrin strands form between the wall of the cuvette and a piston suspended in the sample, the rotational motion gets transferred to the piston, and an electronic amplification system allows the characteristic tracing to be recorded.

Blood samples with anti-coagulant were spun at 3000 rpm for 10 min, supernatants (plasma) were aliquoted into siliconized eppendorf tubes (SafeSeal, PGC Scientifics, USA), frozen in dry ice and stored at -80 °C until use for lipid extraction and measurement of plasma TG/cholesterol.

### Mass spectrometry analysis of lipids extracted from plasma

HPLC-grade acetonitrile, isopropanol, formic acid and ammonium formate were purchased from Sigma or Fisher Scientific (USA). Ultrapure water from Milli-Q purification system (Millipore, USA) was used.

Plasma lipids were extracted by isopropanol [48]. In brief, 10 μL of plasma was mixed with 500 μL of isopropanol, after vortexing and centrifugation (10,000 × g, 5 min, room temperature), the supernatant was retained and 2 μL of which was loaded into mass spectrometer with an auto sampler through a LC system (UPLC H-class, Waters, USA). A HSS T3 column (1.8 μm, 2.1 mm ID × 100 mm, Waters, USA) maintained at 65 °C was used for separation of lipids. The mobile phase A was isopropanol/acetonitrile/formic acid (90:10:0.1, v/v/v) containing 10 mM ammonium formate; the mobile phase B was acetonitrile/water/formic acid (60:40:0.1, v/v/v) containing 10 mM ammonium formate. The UPLC separations were 12 min/sample using the following scheme: 1) 0 min, 60 % B; 2) 10 min, 1 % B; 3) 12 min, 60 % B. All changes were linear with a flow rate of 400 μL/min.

Lipid profile analysis was performed using ESI Q-TOF MS (Xevo G2, Waters, USA). Both the nebulizer and desolvation gases were nitrogen. Typical operating condition was set as follows: capillary voltage 3.0 kV, sampling cone voltage 40 V, desolvation gas temperature 500 °C, source temperature 150 °C, nebulization gas flow 40 L/h, and desolvation gas flow 400 L/h. The m/z range detected was from 50 to 1200. The positive ion MS^E^ mode was used for data acquisition.

### Statistical analysis

Data obtained by MS were processed by Markerlynx XS and EZ info softwares (Waters, USA). Markerlynx XS was used to carry out peak discrimination, filtering, and retention time alignment, yielding >20000 peaks between the retention time of 0–12 min. To investigate global lipids alterations, all observations acquired were integrated and co-analyzed using partial least square-discriminant analysis by EZ info software. The VIP-plot in EZ info software was further carried out to select variables as potential markers for distinguishing patients from controls. The VIP value reflects the influence of every variable on the classification, and is considered to have above-average influence when higher than 1.0. In our work, the variables with a VIP value of >4.0 were highlighted and selected. Their accurate m/z information was used to search the matched lipid from database established by Waters or from LIPID MAPS (http://www.lipidmaps.org). The fragment ions were further utilized for confirmation of the marker. Significance of difference between multiple groups were analyzed by one-way ANOVA by SigmaStat (v3.5), and *P* <0.05 were considered statistically significant.

## Additional file

Additional file 1: Table S1. Blood coagulation analysis of prothrombin time (PT), activated partial thromboplastin time (APTT) and thrombin time (TT) from healthy children (HC) and Kawasaki disease (KD). Data are presented as mean±SD. **Table S2.** Detailed quantitative data and the statistical analysis of 19 TG molecular species contributing to the difference between the three groups. HC: healthy children; BT: fever patients from bacterial infections; KD: Kawasaki disease. **Table S3.** Detailed quantitative data and the statistical analysis of PC and LPC molecular species contributing to the difference between the three groups. HC: healthy children; BT: fever patients from bacterial infections; KD: Kawasaki disease. (DOCX 33 kb)

## Abbreviations

APTT: activated partial thromboplastin time
BT: children with bacterial infections
BTNγ: butyrophilin γ
CRP: C-reaction protein
DIC: disseminated intravascular coagulation
ESI: electrospray ionization
ESR: erythrocyte sedimentation rate
FFA: free fatty acid
HC: healthy children
HPLC: high performance liquid chromatography
IL: interleukin
KD: Kawasaki disease
LPC: lyso-phosphatidylcholine
MS: mass spectrometry
PC: phosphatidylcholine)
PT: prothrombin time
Q-TOF: quadrupole-time of flight
SM: sphingomyelin
TEG: thromroelastography
TG: triglyceride
TNFα: tumor necrosis factor α
TT: thrombin time
