# Supplementary material for: Hypercoagulation and elevation of blood triglycerides are characteristics of Kawasaki disease
Source: Lipids Health Dis. 2015 Dec 30;14:166. doi: 10.1186/s12944-015-0167-2 (PMC4696131; doi:10.1186/s12944-015-0167-2)
Supplement: Additional file 1: Table S1. — Blood coagulation analysis of prothrombin time (PT), activated partial thromboplastin time (APTT) and thrombin time (TT) from healthy children (HC) and Kawasaki disease (KD). Data are presented as mean±SD. Table S2. Detailed quantitative data and the statistical analysis of 19 TG molecular species contributing to the difference between the three groups. HC: healthy children; BT: fever patients from bacterial infections; KD: Kawasaki disease. Table S3. Detailed quantitative data and the statistical analysis of PC and LPC molecular species contributing to the difference between the three groups. HC: healthy children; BT: fever patients from bacterial infections; KD: Kawasaki disease. (DOCX 33 kb) [file 12944_2015_167_MOESM1_ESM.docx]

**Supplementary tables**

Table S1. Blood coagulation analysis of p[rothrombin time](http://dict.cn/prothrombin%20time) (PT), [activated partial thromboplastin time](http://dict.cn/activated%20partial%20thromboplastin%20time) (APTT) and [thrombin time](http://dict.cn/thrombin%20time) (TT) from healthy children (HC) and Kawasaki disease (KD). Data are presented as mean±SD.

|  | HC | KD | Normal Range |
| --- | --- | --- | --- |
| PT, s | 11.1±0.7 | 11.6±0.9 | 9-14 |
| APTT, s | 29.4±3.4 | 30.3±6.2 | 23-38 |
| [TT](http://dict.cn/thrombin%20time), s | 16.8±0.8 | 16.6±6.1 | 15-22 |

Table S2. Detailed quantitative data and the statistical analysis of 19 TG molecular species contributing to the difference between the three groups. HC: healthy children; BT: fever patients from bacterial infections; KD: Kawasaki disease.

|  | mean | | | SD | | | P | | |
| --- | --- | --- | --- | --- | --- | --- | --- | --- | --- |
| ID | HC | BT | KD | HC | BT | KD | HC-BT | HC-KD | BT-KD |
| TG 48:1 | 88.591 | 194.508 | 288.723 | 35.104 | 105.223 | 78.308 | 0.005 | 0.000 | 0.004 |
| TG 48:2 | 326.244 | 475.608 | 515.179 | 80.086 | 79.042 | 118.171 | 0.002 | 0.000 | 0.122 |
| TG 50:1 | 92.236 | 183.797 | 180.708 | 62.236 | 113.680 | 63.427 | 0.038 | 0.001 | 0.362 |
| TG 50:2 | 39.465 | 105.199 | 102.431 | 20.644 | 74.629 | 31.786 | 0.016 | 0.000 | 0.490 |
| TG 50:3 | 113.576 | 177.152 | 197.282 | 53.387 | 61.604 | 38.598 | 0.024 | 0.000 | 0.092 |
| TG 52:1 | 273.060 | 425.899 | 499.962 | 71.675 | 83.713 | 118.471 | 0.001 | 0.000 | 0.016 |
| TG 52:2 | 101.167 | 209.940 | 243.228 | 25.089 | 75.473 | 40.576 | 0.001 | 0.000 | 0.092 |
| TG 52:3 | 95.198 | 200.414 | 251.729 | 20.983 | 72.544 | 42.125 | 0.001 | 0.000 | 0.038 |
| TG 52:4 | 40.267 | 82.701 | 123.711 | 16.771 | 44.466 | 32.619 | 0.015 | 0.000 | 0.014 |
| TG 54:2 | 43.254 | 63.250 | 63.350 | 25.341 | 39.867 | 29.331 | 0.107 | 0.055 | 0.383 |
| TG 54:3 | 19.549 | 47.132 | 45.341 | 9.916 | 34.340 | 38.146 | 0.013 | 0.034 | 0.452 |
| TG 54:4 | 54.454 | 89.102 | 100.966 | 28.428 | 46.532 | 25.378 | 0.050 | 0.002 | 0.199 |
| TG 54:5 | 40.756 | 89.061 | 103.038 | 12.124 | 44.386 | 25.993 | 0.006 | 0.000 | 0.177 |
| TG 54:6 | 14.307 | 25.029 | 42.046 | 10.698 | 17.397 | 18.000 | 0.016 | 0.000 | 0.075 |
| TG 56:6 | 17.218 | 38.222 | 35.103 | 8.940 | 13.705 | 15.390 | 0.002 | 0.001 | 0.456 |
| TG 56:7 | 10.300 | 38.394 | 50.056 | 8.514 | 17.713 | 25.063 | 0.000 | 0.000 | 0.098 |
| TG 56:8 | 4.768 | 18.316 | 30.346 | 2.921 | 11.394 | 20.561 | 0.002 | 0.000 | 0.023 |
| TG 58:8 | 2.556 | 14.726 | 17.976 | 2.060 | 9.176 | 9.202 | 0.001 | 0.000 | 0.272 |
| TG 58:9 | 2.883 | 12.767 | 17.005 | 1.682 | 8.242 | 9.807 | 0.002 | 0.000 | 0.146 |

Table S3. Detailed quantitative data and the statistical analysis of PC and LPC molecular species contributing to the difference between the three groups. HC: healthy children; BT: fever patients from bacterial infections; KD: Kawasaki disease.

|  | mean | | | SD | | | P | | |
| --- | --- | --- | --- | --- | --- | --- | --- | --- | --- |
| ID | HC | BT | KD | HC | BT | KD | HC-BT | HC-KD | BT-KD |
| PC 14:0/18:1 | 22.522 | 21.571 | 12.257 | 11.450 | 7.738 | 8.552 | 0.424 | 0.032 | 0.001 |
| PC 14:0/20:2 | 346.508 | 244.590 | 236.773 | 129.975 | 134.183 | 108.171 | 0.057 | 0.015 | 0.359 |
| PC 14:0/22:4 | 314.185 | 265.817 | 297.643 | 90.257 | 57.382 | 68.219 | 0.033 | 0.347 | 0.167 |
| PC 16:0/20:3 | 71.052 | 29.433 | 32.440 | 37.421 | 21.542 | 15.692 | 0.000 | 0.003 | 0.437 |
| PC 18:1/18:1 | 184.133 | 99.506 | 114.954 | 88.560 | 81.255 | 63.181 | 0.015 | 0.016 | 0.388 |
| PC 18:0/18:1 | 38.660 | 21.418 | 28.068 | 17.033 | 13.634 | 9.399 | 0.003 | 0.052 | 0.143 |
| PC 18:0/20:3 | 34.985 | 12.679 | 15.878 | 17.592 | 7.057 | 6.844 | 0.000 | 0.008 | 0.234 |
| LPC 16:0 | 54.279 | 19.027 | 38.439 | 17.600 | 11.064 | 12.552 | 0.000 | 0.027 | 0.005 |
| LPC 18:0 | 14.792 | 3.205 | 9.133 | 4.018 | 3.755 | 4.698 | 0.000 | 0.010 | 0.007 |
